# Predicting the Function of 4-Coumarate:CoA Ligase (*LJ4CL*1) in *Lonicera japonica*

**DOI:** 10.3390/ijms15022386

**Published:** 2014-02-10

**Authors:** Yuan Yuan, Shulin Yu, Jun Yu, Zhilai Zhan, Minhui Li, Guiming Liu, Xumin Wang, Luqi Huang

**Affiliations:** 1 State Key Laboratory of Dao-di Herbs, National Resource Center for Chinese Materia Medica, Academy of Chinese Medical Sciences, Beijing 100700, China; E-Mails: yuanyuan@icmm.ac.cn (Y.Y.); zzlzhongyi@163.com (Z.Z.); li_minhui@aliyun.com (M.L.); 2 Pharmacy College, Anhui University of Chinese Medicine, Hefei 230038, China; E-Mail: yushulinss@163.com; 3 CAS Key Laboratory of Genome Sciences and Information, Beijing 100029, China; E-Mails: junyu@big.ac.cn (J.Y.); liugm@big.ac.cn (G.L.)

**Keywords:** 4-coumarate:CoA ligase, phenylpropanoid-derived compounds, *Lonicera japonica*, phylogeny

## Abstract

4-Coumarate:CoA ligases (4CLs) are a group of essential enzymes involved in the pathway of phenylpropanoid-derived compound metabolisms; however it is still difficult to identify orthologs and paralogs of these important enzymes just based on sequence similarity of the conserved domains. Using sequence data of 20 plant species from the public databases and sequences from *Lonicera japonica*, we define 1252 adenosine monophosphate (AMP)-dependent synthetase/ligase sequences and classify them into three phylogenetic clades. 4CLs are in one of the four subgroups, according to their partitioning, with known proteins characterized in *A. thaliana* and *Oryza sativa*. We also defined 184 non-redundant sequences that encode proteins containing the GEICIRG motif and the taxonomic distribution of these GEICIRG-containing proteins suggests unique catalytic activities in plants. We further analyzed their transcription levels in *L. japonica* and *L. japonica*. var. chinensis flowers and chose the highest expressed genes representing the subgroups for structure and binding site predictions. Coupled with liquid chromatography-mass spectrometry (LC-MS) analysis of the *L. japonica* flowers, the structural study on putative substrate binding amino acid residues, ferulate, and 4-coumaric acid of the conserved binding-site of *LJ4CL*1 leads to a conclusion that this highly expressed protein group in the flowers may process 4-coumarate that represents 90% of the known phenylpropanoid-derived compounds. The activity of purified crude *LJ4CL*1 protein was analyzed using 4-coumarate as template and high activity indicating that 4-coumarate is one of the substrates of *LJ4CL*1.

## 1. Introduction

4-Coumarate:CoA ligases (4CLs, EC 6.2.1.12) are a group of essential enzymes involved in the phenylpropanoid-derived compound (PDC) pathway, which converts hydroxylated cinnamic acids into their corresponding thioesters [1]. The PDC pathway as well as its branch pathways generates various classes of secondary compounds, including lignin, flavones, flavonols, anthocyanins, isoflavonoids, and furanocoumarins [2]. PDCs, as a group of the ubiquitous plant secondary metabolites, control flower color, pollination, and stress response [3]. In medicinal plants, certain PDCs have important functions, such as anti-inflammatory, anti-tumor, and anti-human immunodeficiency virus activity [4].

Due to the importance of phenylpropanoid-derived products in plants, 4CL, characterized as a member of a large AMP-binding protein family, has been studied extensively for nearly four decades [5]. However it is still difficult to identify them just based on sequence similarity of conserved domains. Although functionality can be deduced from the domain composition of proteins and enzymes [6], detailed domain analysis of 4CLs remain largely unknown. Their first signature domain (Box I) consists of a serine/threonine/glycine (STG)-rich domain followed by a proline/lysine/glycine (PKG) triplet [7], whereas the second signature domain contains a GEICIRG motif (Box II) [8]. The 4CL-catalyzed CoA ester formation takes place via a two-step reaction. In the first step, 4-coumarate and ATP form a coumaroyl-adenylate intermediate with simultaneous release of pyrophosphate. In the second step, the coumaroyl group is transferred to the sulfhydryl group of CoA, and AMP is subsequently released [8]. The mechanism of an adenylate intermediate formation is also common among a number of other enzymes with divergent functions, including luciferases, fatty acyl-CoA ligases, acetyl-CoA ligases, and the specialized domains of peptide synthetase multienzymes. Despite their low overall amino acid sequence identity, similar reaction mechanisms and the presence of conserved peptide motifs are used to classify 4CLs into a superfamily of adenylate-forming enzymes [9]. The relationship of 4CLs with other adenylate-forming enzymes is recently substantiated by a functional analysis of those key 4CL amino acid residues conserved in other adenylate-forming enzymes [10]. Phylogenetic analyses of the superfamily of adenylate-forming enzymes show that 4CL forms a monophyletic plant-specific group more closely related to luciferases rather than to the long-chain acyl-CoA ligases and acetyl-CoA ligases [11]. However, Souza *et al*. [12] reported that acyl-CoA synthetase is related to 4CL, although it encodes a novel fatty acyl-CoA synthetase. An *in silico* analysis revealed that the *Arabidopsis* genome has 14 genes annotated as putative 4-coumarate:CoA ligase isoforms or homologs. Of these genes, only four are catalytically active *in vitro*, with broad substrate specificities [13], and the functions of the others are yet to be characterized.

4CLs often present in multiple isoforms that exhibit distinct substrate specificity and coincide with specific metabolic functions (Figure S1). Substrate of 4CLs include sinapic acid, 5-hydroxyferulate, ferulic acid, caffeic acid, 4-coumarate, and *trans-*cinnamic acid. Three 4CL isozymes from *Sorbus aucuparia* L. prefer 4-coumaric acid over cinnamic acid in the spectrophotometric assays, but fail to utilize benzoic acid in radioisotopic assays [14]. Allina *et al*. [15] confirmed that multiple 4CL isoforms present in poplar tissues. However, there has been no evidence to support the differences in substrate-utilization profiles of the partially purified native 4CL isoforms or of the two isoforms expressed in the recombinant forms. Three of the 4CLs from the bryophyte *Physcomitrella patens* display similar substrate utilization profiles with high catalytic efficiency towards 4-coumarate, but similar efficiency with cinnamate as the substrate to those with caffeate and ferulate [16]. All are efficiently activated by 4CLs from various sources except sinapate [17]. Recombinant *Ocimum sanctum* 4CL showed the highest activity with *p*-coumaric acid, followed by ferulic, caffeic, and *trans-*cinnamic acids [18]. One of the *Petunia* 4CLs has broad substrate specificity and represents a *bona fide* 4CL, whereas the other is a cinnamate:CoA ligase [19]. The crystal structures of 4CLs from *Arabidopsis thaliana* [20] and *Populus tomentosa* [21] have already been reported. Information regarding 4CL specificity may facilitate predicting substrate preference for the characterization of 4CL-like proteins [22]. Divergent substrate preference also affects the expression of 4CL genes [1]. A differential transcription pattern of each 4CL, in various organs and tissues, as well as distinct temporal patterns of expression, has been observed during flower and fruit development of raspberry [23]. The controlled silencing of *At4CL1* and *At4CL2* alter the lignocellulose composition of *Arabidopsis* without affecting its stem growth [24]. Likewise, severe suppression of 4CLs in the coniferous *Pinus radiata* substantially affects plant phenotype and results in dwarfed plants [25].

The major active PDCs in *Lonicera japonica* are flavones and flavonols, including chlorogenic acid (CGAs) and luteoloside [26–28]. In this study, we aim to determine the characteristics and function of 4CLs in *L. japonica*. Recently, a number of 4CLs have been characterized in *A. thaliana* and *Oryza sativa* based on transcriptomic studies [29]. Here we report the identification and characterization of *LJ4CL*s and propose the relationship of *LJ4CL* function and the related active compounds in *L. japonica*, based on expression data [30], protein structure analysis, and substrate characterization.

## 2. Results and Discussion

### 2.1. Global Phylogeny and Duplication of AMP-Binding Proteins

Using Pfam (AMP-binding enzyme PF00501) and Interpro (IPR000873 and IPR020845), as well as information from public genome databases and our own transcriptome databases of *L. japonica*, we gathered 1252 non-redundant sequences that encode AMP-binding proteins from 20 different species, representing a diverse taxonomic background (Table S1). The result shows that AMP-binding proteins are widely distributed among bacteria, fungi, animals, and plants. Among 1252 sequences, 146 putative AMP-binding protein sequences are identified in *A. lyrata* as compared to 46 and 10 AMP-binding proteins in *Culex quinquefasciatus* and *Escherichia coli*, respectively (Table 1).

We classified all AMP-binding protein sequences into three clusters, where 82% of them are in Cluster 1. The gymnospermae species are all found in cluster 1, whereas pteridophyta, algae, monocotyledoneae, and dicotyledoneae are divided into both Clusters 1 and 2. A few sequences from *A. lyrata* and *L. japonica Thunb* var. chinensis (Wats.) are in Cluster 3. Cluster 1 has four subgroups. According to the known function of the proteins in *A. thaliana* and *O. sativa*, we speculate that long chain acyl-CoA synthase (ACS) belongs to Subgroup 1 and that acyl-acting enzyme (AAE), *o*-succinylbenzoate-CoA ligase, and benzoate-CoA ligase are in Subgroup 2. 4CL is expected to be in Subgroup 3, and Subgroup 4 includes acyl-CoA and malonyl-CoA synthases. The predicted 4CL group is the same as true 4CL enzymes from genome-wide analysis of a land plant-specific acyl:coenzymeA synthetase (ACS) gene family in *Arabidopsis*, poplar, rice, and *Physcomitrella* [31] and 4CL-like. From the Neighbor-Joining trees, we also found that the gymnospermae sequences are only clustered in Subgroup 3, but the algal sequences are not (Figure S2).

### 2.2. Global Phylogeny and Duplication of GEICIRG-Containing Proteins

In the species of which the genome is completely known, there are 184 non-redundant sequences that encode GEICIRG-containing proteins, which are unique to Plantae, including gymnospermae, algae, bryophyte, pteridophyta, and angiospermae (Table 2). The GEICIRG motif is absolutely conserved in all 4CLs, and its central cysteine residue is suggested to be directly involved in catalysis [8], and the participation of a cysteine residue in catalysis has also been observed for other adenylate-forming enzymes [32].

To obtain a global view of the phylogenetic relationships among GEICIRG-containing proteins, we first constructed an NJ tree, based on their AMP-dependent synthetase/ligase domain sequences (Figure S3). These sequences clustered together with a strong bootstrap support and four clades are clearly distinguishable, including 4CLs, two ASCs, and one AAE protein as subgroups, based on the known functions of the proteins in *A. thaliana* and *O. sativa*.

The phylogenetic reconstructions also revealed that subsequent duplication of proteins containing the GEICIRG motif occurred in different lineages. Analysis on these clades—4CLs, ACSs, and AAE in dicotyledoneae, monocotyledoneae, pteridophyta, bryophyte, algae, and gymnospermae—suggests that gene duplication occurred among the GEICIRG-containing proteins prior to the divergence of Angiospermae and Pteridophyta. In the NJ tree, representatives of 4CL and AAE are classified into two ASC clades. The major sequences in the AAE clade (Cluster 2) are from Pteridophyta and Dicotyledoneae, where only one copy is found in *Sorghum bicolor*.

The phylogeny based on AMP-dependent synthetase/ligase domain sequences (Figures S4 and S5) revealed that ACS proteins are classified into two clusters (1 and 4). In dicots, the lineage-specific duplication events in Cluster 1 are similar to those in Cluster 4; however, Cluster 4 has more duplication events than Cluster 1 in monocots. In cluster 1, two ACS copies are paralogous in *Chlamydomonas reinhardtii*, indicating that duplication occurred early in angiosperm evolution. Three ACS copies are also present in *Selaginella moellendorffii* and are separated by angiosperm sequences, which are related to ancient gene duplication.

**Figure 1.**
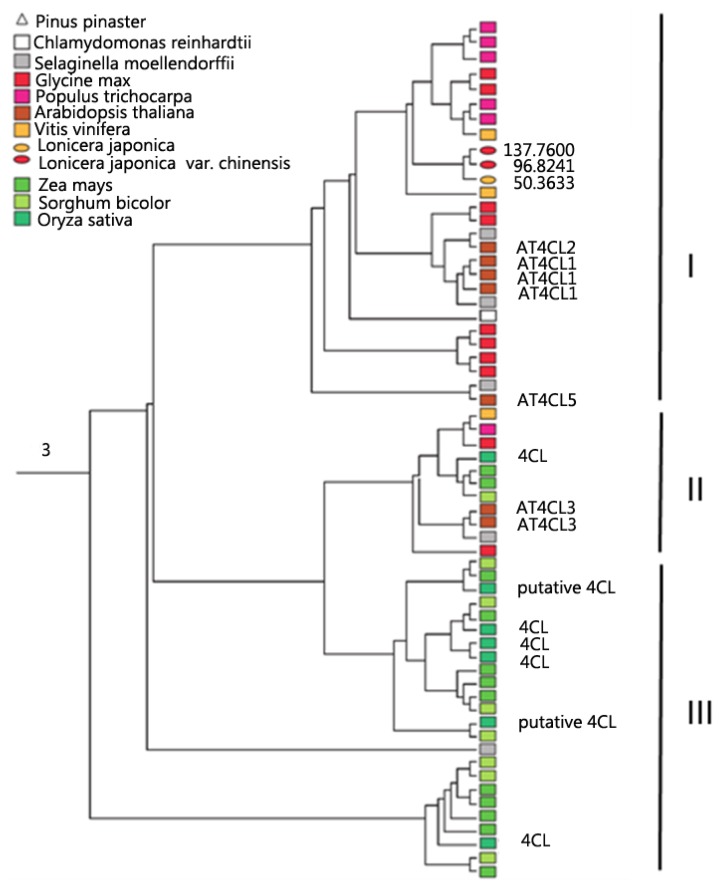
Phylogeny and expression of 4CL sequences in Cluster 3. A neighbor-joining tree containing 62 sequences was generated based on the AMP-dependent synthetase/ligase domain sequences. A bootstrap value of 1000 replications was applied. The RPKM value of sequences in flowers of *Lonicera japonica* is shown.

**Figure 2.**
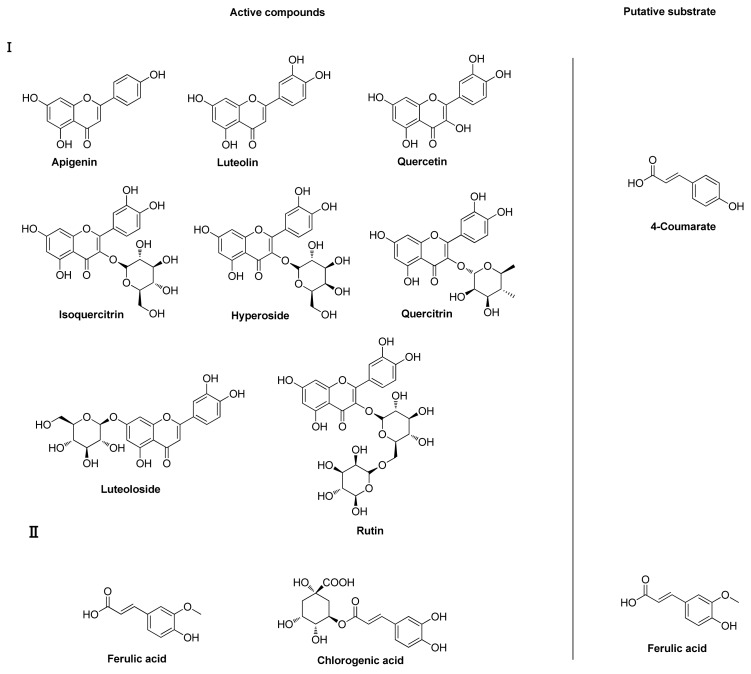
PDCs in *Lonicera japonica*.

The most abundant group, containing 4CLs (Cluster 3), shares the GEICIRG-motif. The phylogeny based on AMP-dependent synthetase/ligase domain sequences (Figure 1) revealed distinct and highly supported clades that group the 4CL proteins into: (1) Dicotyledoneae; (2) Mixed group; and (3) Monocotyledoneae. Apart from the early duplication of 4CLs during the evolution of higher plants, successive duplications must have occurred among eudicots and monocots as additional copies are present in *Arabidopsis* (7 copies) and rice (7 copies). Ten and 12 copies are also evident in *Glycine max* and in *Zea mays*, respectively (Figure 2). The high occurrence of duplication events are attributable to frequent genome duplications in angiosperm evolution.

### 2.3. Expression of GEICIRG-Containing Proteins in *L. japonica* Flowers

We analyzed the transcription of the GEICIRG-containing proteins based on our own data. In Cluster 1, the GEICIRG-containing proteins are clustered into two groups and the first group contains two pairs of orthologs. In the other group, the paralogs of *L. japonica* are found to be expressed at low levels, and their average Reads Per Kilo bases per Million reads (RPKM) is 22.91, which is lower than that of the first group. Although there are a similar number of orthologs in *L. japonica* and *L. japonica* var. chinensis, the collective average RPKM value of the GEICIRG-containing paralogs is 1.87-fold higher in *L. japonica* than that in *L. japonica* var. chinensis. A similar trend is found in Cluster 2 and other results are summarized in Table 3.

### 2.4. Substrate-Binding Diversity in the Expressed GEICIRG-Containing Proteins in *L. japonica*

We examined the structure of four GEICIRG-containing proteins in *L. japonica* based on their expressions—the most highly expressed genes in each cluster (Figure S6; Table S2). Only six conserved residues (L-243, H-247, S-316, Y-342, T-345, and E-346, in LJ4CL1) were found, and the T-345 residue has the highest frequency over all others. Another high-frequency residue, M-332, belonging to the B-subdomain (adenylation domain), is relatively conserved [40] but is not found in LJACS2. Thus, T-345 may be the conserved residue responsible for the function of adenylation domain in *L. japonica*.

Highly expressed LJ4CL1 is found in *L. japonica* bud [30], and it has the following putative substrate binding residues: I-196, Y-197, G-317, G-343, P-349, V-350, and L-351. The substrate-binding residues Y-236, G-306, G-331, P-337, and V-338 in 4CL1 of *P. tomentosa* are identified, based on their crystal structures, as well as mutagenesis and enzymatic activity studies. The Y residues may relate to Pt4CL1 activity against caffeic and ferulic acids, but not 4-coumaric acid. Similarly, the G residue may relate to Pt4CL1 activity against 4-coumaric and caffeic acids [21]. However, Schneider *et al*. [22] also reported that 12 amino acid residues, I-252, Y-253, N-256, M-293, K-320, G-322, A-323, G-346, G-348, P-354, V-355, and L-356, form the substrate specificity code of At4CL. The N-256 residue is located at a distance of 3.1 Å from the hydrogen atom of the 4-hydroxyl group of caffeic acid. The residues M-293 and K-320 formed a clamp structure around the 3-hydroxyl group of caffeate. However, the residues N-256, M-293, and K-320 do not seem to have the corresponding residues in LJ4CL1.

Our results suggest that ferulic acid and 4-coumaric acid are candidate substrates of LJ4CL1. To verify our prediction, we analyzed the PDCs of *L. japonica* flowers using LC-MS. Ten compounds, namely, chlorogenic acid, ferulic acid, rutin, hyperoside, isoquercitrin, luteoloside, quercitrin, luteolin, quercetin, and apigenin, are identified. Considering the transferase reaction that couples quinic acid to cinnamic acid derivatives is reversible, the CGAs are a storage form of cinnamic acid derivatives, and are considered as intermediates in the lignin biosynthetic pathway [41,42]. Based on their chemical structure, the intermediates are classified into two groups. The putative substrates of these two groups are 4-coumarate and ferulic acid, consistent with the putative function of LJ4CL1.

I-196, Y-197, G-317, and G-343 are also observed in LJACS1 and LJACS2, and their frequencies are lower than those in LJ4CL. P-349, V-350, and L-351 are not found in LJACSs and LJAAE, and they may be conserved residues related to 4-coumarate. The size of the binding pocket is most important in determining the substrate specificities of *P. tomentosa* 4CLs [21]. Our results suggest that the diversity of the residues of the binding-site controls the enzymatic function, although they have the same conserved catalytic motif. Ninety percent of the substrates for the known PDCs in *L. japonica* are 4-coumarate, and the substrate specificity may be related to the conserved residues in LJ4CLs.

To confirm our suspect, LJ4CL1 protein was expressed in *E. coli*. The activity of crude LJ4CL1 protein was analyzed using 4-coumarate as template and high activity (19.36 U μg^−1^ protein^−1^) was observed, indicating that 4-coumarate is one of the substrates of LJ4CL1.

## 3. Experimental Section

### 3.1. Classification of AMP-Dependent Synthetase/Ligase Sequences and GEICIRG-Motif Proteins

We searched for the AMP-dependent synthetase/ligase sequences of twenty-one species (Table S1) using the pfam [43] and InterPro databases. These species include one animal, one bacterium, two fungi, two algae, three gymnospermae, two pteridophyta, seven dicotyledoneae, and three monocotyledoneae. We compared the sequences against the sequence “GEICIRG” with an *e*-value cut-off below *e*^−20^ using BlastP (protein-protein BLAST) [44] to determine the GEICIRG-containing proteins from the best reciprocal hits.

### 3.2. Phylogeny of AMP-Dependent Synthetase/Ligase Sequences and GEICIRG-Motif Proteins

We used the AMP-dependent synthetase/ligase domain of AMP-dependent synthetase/ligase sequences and GEICIRG-motif proteins to construct neighbor-joining trees using Mega 5.0 [45] and ClustalW2 [46], respectively, with a bootstrap value of 1000 replicates. In addition, we reconciled preliminary trees by setting the bootstrap value greater than 50% to yield a more credible consensus tree.

### 3.3. Indentification of Orthologs and Paralogs

To identify orthologs, we performed an all-against-all sequence comparison using BLAST with an *e*-value cut-off below *e*^−20^. The orthologs were then determined based on the best reciprocal hits [47]. We implemented a more stringent criterion that the alignment length percentage against the longer protein must be above 80%.

### 3.4. Gene Expression Analyses

The gene expression profiling of *L. japonica* flowers was performed in a previous work [30]. The expression level was normalized with total mapped reads and the contig length, similar to the reads per kilobase of exon model per million mapped reads (RPKM) method [48]. The RPKM value for each transcript was calculated as the number of reads per kilobase of the transcript sequence per million mapped reads [49].

### 3.5. Protein Structure and Binding Site Prediction

The three-dimensional protein structures were predicted from the amino acid sequences using the online version of I-TASSER [50]. Based on the C-score and TM-score, the top ten models were predicted and the structural analogs with similar binding sites were identified. All of the predicted binding site residues in the model were summarized. The diversity of the predicted binding site residues of four proteins was analyzed.

### 3.6. LC-MS Analysis of *L. japonica* Flowers

Dried *L. japonica* flowers (medicinal materials) were separately comminuted with a miller. Each solid sample (40 mesh, 0.50 g) was accurately weighed, and extracted with 50 mL of 70% aqueous ethanol with ultrasonication for 30 min. The extract was cooled to 25 ºC, diluted to 50 mL with 70% aqueous ethanol, and filtered with a 0.45 μm Millipore filter membrane. Then, 10 μL of the filtrate was injected into the liquid chromatography–mass spectrometry (LC-MS) system (Agilent RRLC/Agilent ion trap 6320, Agilent, Santa Clara, CA, USA) for analysis (Figure S7). The LC-MS/MS systems were set to a 1.0 mL/min flow rate and performed in an Agilent TC-C_18_ reserved-phase column (5 μm, 250 mm × 4.6 mm). The mobile phases consisted of deionized water-formic acid (99:1, *v*/*v*) and methanol. The elution conditions were same as the high-performance liquid chromatography (HPLC, Agilent, Santa Clara, CA, USA) conditions used in a previous work [30]. The detection wavelength was set to 242 nm, and the column temperature was maintained at 25 ºC. All standard compounds were purchased from the National Institutes for Food and Drug Control, Beijing, China.

### 3.7. Expression of 4CL Protein in *E.coli* and Enzyme Activity Assay

The open reading frame (ORF) of LJ4CL was cloned into the expression vector pGEX-4T-1 and transformed into Transetta (DE3) chemically competent cells (Beijing TransGen Biotech Co., Ltd., Beijing, China), respectively. The vector pGEX-4T-1 (+) allows inframe cloning of PCR products resulting in a GST-tag attached at the *N*-terminal end of the recombinant protein. Expression of the recombinant protein was induced by adding isopropyl-β-d-1-thiogalactopyranoside (IPTG) and cells were harvested at 9 h. The activity of 4CL was analyzed according to Voo *et al*. [51]. The 1 mL reaction mixture contained 50 μL crude enzyme, 0.2 mM 4-coumarate, 0.8 mM ATP, 7.5 mM MgCl_2_, and 38 M CoA in 100 mM Tris-HCl buffer (pH 7.5). One unit of 4CL was defined as the amount of enzyme that causes a decrease in A333 of 0.01 units min^−1^. Protein concentration in the extracts was determined using the Lowry method [52].

## 4. Conclusions

4CLs form an important enzyme family for the phenylpropanoid-derived pathway in plants. Our analysis on AMP-binding and GEICIRG-containing proteins from the genome and transcript sequences of 19 species; including an in-house generated dataset containing 40,000 transcript scaffolds of *L. japonica*; allowed us to further exploit 4CL structural (domain and motif) features and validate structural predictions based on chemical assays. We also propose the putative substrate-binding residues of LJ4CLs and defined the major substrate of the PDC pathway in *L. japonica*. Our study paves a way for further studies on 4CLs and their related metabolic pathways in medicinal plants.

## Supplementary Information



## Figures and Tables

**Table 1.** Copy number of AMP-binding domain in 20 species.

Kingdom Group		Class	Clusters *	Number of copies											
			Species	1	1-1	1-2	1-3	1-4	2	2-1	2-2	2-3	2-4	3	Total

Animal			*Culex quinquefasciatus*	39	5	5	27	2	6	1	4	1	0	1	46
Bacteria			*Escherichia coli*	5	0	3	2	0	3	0	2	1	0	2	10
Fungus			*Penicillium marneffei*	20	3	3	12	2	25	3	7	15	0	5	50
			*Aspergillus nidulans*	32	2	8	17	5	28	3	5	19	1	1	61

Plant	Algae		*Chlamydomonas reinhardtii*	18	11	2	0	5	9	0	8	0	1	0	27
	Bryophyte		*Physcomitrella patens*	43	8	16	14	5	2	0	0	1	1	0	45
	Pteridophyta		*Selaginella moellendorffii*	70	15	26	26	3	14	3	9	2	0	0	84

	Gymnospermae		*Pinus taeda*	4	0	0	4	0	0	0	0	0	0	0	4
			*Larix gmelinii*	18	0	0	18	0	0	0	0	0	0	0	18
			*Pseudotsuga menziesii*	17	0	0	17	0	0	0	0	0	0	0	17

	Angiospermae	Dicotyledoneae	*Glycine max*	99	30	29	35	5	12	5	6	1	0	0	111
			*Populus trichocarpa*	71	21	24	23	3	8	3	4	1	0	0	79
			*Arabidopsis thaliana*	48	13	17	16	2	6	0	5	1	0	0	54
			*Arabidopsis lyrata*	146	24	0	122	0	25	8	15	2	0	1	172
			*Vitis vinifera*	34	8	9	14	3	6	1	4	1	0	0	40
			*Lonicera japonica*	103	43	36	17	7	14	0	12	2	0	0	117

			*Lonicera japonica* var. chinensis	73	33	20	16	4	12	0	10	2	0	1	86

		Monocotyledoneae	*Zea mays*	82	23	21	35	3	15	3	9	3	0	0	97
			*Sorghum bicolor*	48	16	13	17	2	10	3	6	1	0	0	58
			*Oryza sativa*	60	31	12	16	1	16	3	9	4	0	0	76

Total															1252

* Clusters were showed in Figure S2.

**Table 2.** Copy number of containing-GEICIRG protein.

		Clusters *	Number of copies				Total	Genome size	Reference

			ACS	AAE	4CL	ACS			
		
Species			1	2	3	4		M	
Gymnospermae		*Pinus pinaster*	0	0	1	0	1		
Algae		*Chlamydomonas reinhardtii*	2	0	0	1	3		
Bryophyte		*Physcomitrella patens*	0	3	12	0	15		
Pteridophyta		*Selaginella moellendorffii*	3	2	5	6	16		

Angiospermae	Dicotyledoneae	*Glycine max*	3	7	10	4	24	1100	[33]
		*Populus trichocarpa*	5	1	6	2	14	485	[34]
		*Arabidopsis thaliana*	2	1	7	5	15	135	[35]
		*Vitis vinifera*	2	2	3	2	9	505	[36]
		*Lonicera japonica*	5	4	1	5	15		
		*Lonicera japonica* var. chinensis	4	2	2	3	11	~800	Our group

	Monocotyledoneae	*Zea mays*	3	0	12	8	23	2300	[37]
		*Sorghum bicolor*	2	1	8	4	15	730	[38]
		*Oryza sativa*	6	0	7	10	23	467	[39]

Total			37	23	74	50	184		

* Clusters were showed in Figure S3. ACS, long chain acyl-CoA synthase; AAE, Acyl-acting enzyme/*o*-succinylbenzoate-CoA ligase/benzoate-CoA ligase; 4CL, 4-coumarate:CoA ligase.

**Table 3.** Copy and RPKM of contained GEICIRG protein in *Lonicera japonica*.

Cluster *	Varieties	*Lonicera japonica*	*Lonicera japonica* var. chinensis	Total

	Copy and RPKM			
1	Number of copies	5	4	9
	RPKM	320.4257	171.5426	491.9683

2	Number of copies	4	2	6
	RPKM	250.9583	306.7299	557.6882

3	Number of copies	1	2	3
	RPKM	50.3633	234.6021	284.9654

4	Number of copies	5	3	8
	RPKM	292.3302	236.8353	529.1655

* Clusters were shown in Figure S3.

## References

[1] Hu W.J., Kawaoka A., Tsai C.J. (1998). Compartmentalized expression of two structurally and functionally distinct 4-coumarate:CoA ligase genes in aspen (*Populus tremuloides*). Proc. Natl. Acad. Sci. USA.

[2] Hamberger B., Hahlbrock K. (2004). The 4-coumarate:CoA ligase gene family in *Arabidopsis thaliana* comprises one rare, sinapate-activating and three commonly occurring isoenzymes. Proc. Natl. Acad. Sci. USA.

[3] Ververidis F., Trantas E., Douglas C. (2007). Biotechnology of flavonoids and other phenylpropanoid-derived natural products. Part I: Chemical diversity, impacts on plant biology and human health. Biotechnol. J.

[4] Blach-Olszewska Z., Jatczak B., Rak A. (2008). Production of cytokines and stimulation of resistance to viral infection in human leukocytes by *Scutellaria baicalensis* flavones. J. Interf. Cytokine Res.

[5] Kaneko M., Ohnishi Y., Horinouchi S. (2003). Cinnamate: Coenzyme A ligase from the filamentous bacterium *Streptomyces coelicolor* A3(2). J. Bacteriol.

[6] Cohen-Gihon I., Nussinov R., Sharan R. (2007). Comprehensive analysis of co-occurring domain sets in yeast proteins. BMC Genomics.

[7] Bairoch A. (1991). Prosite—A dictionary of sites and patterns in proteins. Nucleic Acids Res.

[8] Becker-Andre M., Schulze-Lefert P., Hahlbrock K. (1991). Structural comparison, modes of expression, and putative cis-acting elements of the two 4-coumarate:CoA ligase genes in potato. J. Biol. Chem.

[9] Fulda M., Heinz E., Wolter F.P. (1994). The fadd gene of *Escherichia coli* K12 is located close to *rnd* at 39.6 min of the chromosomal map and is a new member of the amp-binding protein family. Mol. Gen. Genet.

[10] Stuible H.P., Buttner D., Ehlting J. (2000). Mutational analysis of 4-coumarate:CoA ligase identifies functionally important amino acids and verifies its close relationship to other adenylate-forming enzymes. FEBS Lett.

[11] Cukovic D., Ehlting J., VanZiffle J.A. (2001). Structure and evolution of 4-coumarate:Coenzyme A ligase (4CL) gene families. Biol. Chem.

[12] Souza C.D., Kim S.S., Koch S. (2009). A novel fatty acyl-coA synthetase is required for pollen development and sporopollenin biosynthesis in *Arabidopsis*. Plant Cell.

[13] Costa M.A., Bedgar D.L., Moinuddin S.G.A. (2005). Characterization *in vitro* and *in vivo* of the putative multigene 4-coumarate:CoA ligase network in *Arabidopsis*: Syringyl lignin and sinapate/sinapyl alcohol derivative formation. Phytochemistry.

[14] Gaid M.M., Scharnhop H., Ramadan H. (2011). 4-Coumarate:CoA ligase family members from elicitor-treated Sorbus aucuparia cell cultures. J. Plant Physiol.

[15] Allina S.M., Pri-Hadash A., Theilmann D.A. (1998). 4-coumarate:Coenzyme A ligase in hybrid poplar—Properties of native enzymes, cDNA cloning, and analysis of recombinant enzymes. Plant Physiol.

[16] Silber M.V., Meimberg H., Ebel J. (2008). Identification of a 4-coumarate:CoA ligase gene family in the moss *Physcomitrella patens*. Phytochemistry.

[17] Hamada K., Nishida T., Yamauchi K. (2004). 4-Coumarate:Coenzyme A ligase in black locust (*Robinia pseudoacacia*) catalyses the conversion of sinapate to sinapoyl-CoA. J. Plant Res.

[18] Rastogi S., Kumar R., Chanotiya C.S. (2013). 4-Coumarate:CoA ligase partitions metabolites for eugenol biosynthesis. Plant Cell Physiol.

[19] Klempien W., Kaminaga Y., Qualley A. (2012). Contribution of CoA ligases to benzenoid biosynthesis in petunia flowers. Plant Cell.

[20] Morita H., Mori T., Wanibuchi K. (2011). Crystallization and preliminary X-ray analysis of 4-coumarate:CoA ligase from *Arabidopsis thaliana*. Acta Crystallogr. Sect. F-Struct. Biol. Cryst. Commun.

[21] Hu Y.L., Gai Y., Yin L. (2010). Crystal structures of a populus tomentosa 4-coumarate:CoA ligase shed light on its enzymatic mechanisms. Plant Cell.

[22] Schneider K., Hovel K., Witzel K. (2003). The substrate specificity-determining amino acid code of 4-coumarate:CoA ligase. Proc. Natl. Acad. Sci. USA.

[23] Kumar A., Ellis B.E. (2003). 4-Coumarate:CoA ligase gene family in *Rubus idaeus*: cDNA structures, evolution, and expression. Plant Mol. Biol.

[24] Yang J.M., Chen F., Yu O. (2011). Controlled silencing of 4-coumarate:CoA ligase alters lignocellulose composition without affecting stem growth. Plant Physiol. Biochem.

[25] Wagner A., Donaldson L., Kim H. (2009). Suppression of 4-coumarate-CoA ligase in the coniferous gymnosperm *Pinus radiata*. Plant Physiol.

[26] Chen C.Y., Qi L.W., Li H.J., Li P., Yi L., Liang H., Tang M.D. (2007). Simultaneous determination of iridoids, phenolicacids, flavonoids, and saponins in *FlosLonicerae* and *FlosLonicerae Japonicae* by HPLC-DAD-ELSDcoupled with principal component analysis. J. Sep. Sci.

[27] Qi L.W., Chen C.Y., Li P. (2009). Structural characterization and identification of iridoidglycosides, saponins, phenolic acids and flavonoids in Flos Lonicerae Japonicae by a fast liquidchromatographymethod with diode-array detection and time-of-flightmass spectrometry. Rapid Commun. Mass Spectrom.

[28] Peng Y.Y., Liu F.H., Ye J.N. (2005). Determination of phenolic acids and flavones in *Lonicerajaponica thumb* by capillary electrophoresis with electrochemical detection. Electroanalysis.

[29] Kushwaha H.R., Singh A.K., Sopory S.K. (2009). Genome wide expression analysis of CBS domain containing proteins in *Arabidopsis thaliana* (L.) Heynh and *Oryza sativa* L. reveals their developmental and stress regulation. BMC Genomics.

[30] Yuan Y., Song L., Li M. (2012). Genetic variation and metabolic pathway intricacy govern the active compound content and quality of the Chinese medicinal plant *Lonicera japonica* thunb. BMC Genomics.

[31] Souza C.A., Barbazuk B., Ralph S.G. (2008). Genome-wide analysis of a land plant-specific acyl:coenzyme A synthetase (ACS) gene family in *Arabidopsis*, poplar, rice and *Physcomitrella*. New Phytol.

[32] Stein T., Vater J., Kruft V. (1996). The multiple carrier model of nonribosomal peptide biosynthesis at modular multienzymatic templates. J. Biol. Chem.

[33] Huang S., Li R., Zhang Z. (2010). Genome sequence of the palaeopolyploid soybean. Nature.

[34] Tuskan G.A., Difazio S., Jansson S. (2006). The genome of black cottonwood, *Populus trichocarpa* (Torr. & Gray). Science.

[35] (2000). The *Arabidopsis* Genome Initiative. Analysis of the genome sequence of the flowering plant *Arabidopsis thaliana*. Nature.

[36] Jaillon O., Aury J.M., Noel B. (2007). The grapevine genome sequence suggests ancestral hexaploidization in major angiosperm phyla. Nature.

[37] Schnable P.S., Ware D., Fulton R.S. (2009). The B73 maize genome: Complexity, diversity, and dynamics. Science.

[38] Paterson A.H., Bowers J.E., Bruggmann R. (2009). The *Sorghum bicolor* genome and the diversification of grasses. Nature.

[39] Yu J., Hu S., Wang J. (2002). A draft sequence of the rice genome (*Oryza sativa* L. ssp. indica). Science.

[40] Khurana P., Gokhale R.S., Mohanty D. (2010). Genome scale prediction of substrate specificity for acyl adenylate superfamily of enzymes based on active site residue profiles. BMC Bioinforma.

[41] Aerts R.J., Baumann T.W. (1994). Distribution and utilization of chlorogenic acid in coffea seedlings. J. Exp. Bot.

[42] Schoch G., Goepfert S., Morant M. (2001). CYP98A3 from *Arabidopsis thaliana* is a 3′-hydroxylase of phenolic esters, a missing link in the phenylpropanoid pathway. J. Biol. Chem.

[43] Bateman A., Birney E., Durbin R. (2000). The Pfam protein families database. Nucleic Acids Res.

[44] Altschul S.F., Madden T.L., Schaffer A.A. (1997). Gapped BLAST and PSI-BLAST: A new generation of protein database search programs. Nucleic Acids Res.

[45] Tamura K., Peterson D., Peterson N. (2011). MEGA5: Molecular evolutionary genetics analysis using maximum likelihood, evolutionary distance, and maximum parsimony methods. Mol. Biol. Evol.

[46] Jeanmougin F., Thompson J.D., Gouy M. (1998). Multiple sequence alignment with Clustal x. Trends Biochem. Sci.

[47] Tatusov R.L., Koonin E.V., Lipman D.J. (1997). A genomic perspective on protein families. Science.

[48] Robinson M.D., Oshlack A. (2010). A scaling normalization method for differential expression analysis of RNA-seq data. Genome Biol.

[49] Mortazavi A., Williams B.A., Mccue K. (2008). Mapping and quantifying mammalian transcriptomes by RNA-Seq. Nat. Methods.

[50] Zhang Y. (2008). I-TASSER server for protein 3D structure prediction. BMC Bioinforma.

[51] Voo K.S., Whetten R.W., O’Malley D.M., Sederoff R.R. (1995). 4-Coumarate: Coenzyme A ligase from loblolly pine xylem (isolation, characterization, and complementary DNA cloning). Plant Physiol.

[52] Lowry O.H., Rosebrough N.R., Farr A.L., Randall R.J. (1951). Protein measurement with the Folinphenol reagent. J. Biol. Chem.

