# Supplementary material for: Predicting the Function of 4-Coumarate:CoA Ligase (LJ4CL1) in Lonicera japonica
Source: Int J Mol Sci. 2014 Feb 10;15(2):2386–99. doi: 10.3390/ijms15022386 (PMC3958857; doi:10.3390/ijms15022386)
Supplement: Supplementary file 1 [file ijms-15-02386-s001.pdf]

Supplementary Information

Table S1. Sequences from twenty-one species in this study.

Species	Genome Database
Arabidopsis lyrata	http://www.phytozome.net/
Arabidopsis thaliana	http://www.phytozome.net/
Aspergillus nidulans FGSC A4	http://www.broad.mit.edu/annotation/fgi
Chlamydomonas reinhardtii	http://www.ncbi.nlm.nih.gov/
Culex quinquefasciatus	http://www.vectorbase.org/Culex_quinquefasciatus/Info/Index
Escherichia coli str. K-12 substr. W3110	http://www.broad.mit.edu
Glycine max	http://www.phytozome.net/
Larix gmelinii	http://dendrome.ucdavis.edu/
Lonicera japonica Thunb	Database in our group
Lonicera japonica Thunb. var. *chinensis* (Wats.)	Database in our group
Oryza sativa	http://www.phytozome.net/
Penicillium marneffei ATCC 18224	http://fungalgenomes.org/data/PEP/
Pinus pinaster	http://dendrome.ucdavis.edu/
Pinus taeda	http://dendrome.ucdavis.edu/
Populus trichocarpa	http://www.phytozome.net/
Pseudotsuga menziesii	http://dendrome.ucdavis.edu/
Selaginella moellendorffii	http://www.phytozome.net/
Sorghum bicolor	http://www.phytozome.net/
Vitis vinifera	http://www.phytozome.net/
Volvox carteri	http://www.ncbi.nlm.nih.gov/
Zea mays	http://www.phytozome.net/

Table S2. Putative binding-site residues in the model *Lonicera japonica*.

LJACS1	LJAAE	LJ4CL	LJACS2
T (151,2) #	T (169, 3)	S (199,1)	M (228, 1)
S (152,2)	S (170,2)	S (200,1)	T (230, 2)
	G (171,1)		
	T (172,1)		
	V (179,1)	V (209,1)	
N (171,2)	S (189,1)	S (219,2)	S (231, 2)
	I (193,1)	Q (223,1)	
L (191,10)	L (209,1)	L (243,1)	L (250, 1)
P (192,10)	P (210,1)	P (244,1)	
A (194,1)			A (278, 1)
H (195,7)	H (213,7)	H (247,5)	H (279, 5)
I (196,5)	V (214,7)	** I (248,6)	I (280, 5)
Y (197,3)	H (215,7)	** Y (249,6)	F (281, 3)
E (198,1)		S (250,1)	
R (199,1)			R (283, 1)
A (200,3)			V (284, 2)
N (201,1)	A (219,6)	I (253,6)	I (285, 1)
C (235,2)	T (257,2)	P (288,2)	C (319, 2)
V (237,1)	V (259,1)	V (290,1)	A (320, 1)

Table S2. *Cont.*

LJACS1	LJAAE	LJ4CL	LJACS2
V (304,1)	R (285,1)	M (315,3)	L (391, 2)
S (305,3)	S (286,3)	S (316,3)	S (392, 3)
G (306,6)	C (287,7)	* G (317,6)	G (393, 6)
A (307,8)	S (288,8)	A (318,7)	A (394, 7)
S (308,5)	A (289,6)	A (319,4)	A (395, 5)
P (309,4)	S (290,5)	P (320,3)	P (396, 5)
V (327,1)	L (308,1)	G (339,2)	L (414, 1)
E (328,4)	E (309,4)	Q (340,5)	Q (415, 6)
G (329,7)	A (310,8)	* G (341,6)	G (416, 6)
Y (330,5)	Y (311,7)	Y (342,4)	Y (417, 5)
G (331,9)	A (312,8)	* G (343,8)	G (418, 8)
M (332,8)	M (313,10)	M (344,7)	L (419, 7)
T (333,8)	T (314,9)	T (345,7)	T (420, 7)
E (334,1)	E (315,2)	E (346,1)	E (421, 1)
S (336,4)	T (317,3)	M (348,4)	C (423, 3)
C (337,4)	H (318,4)	* P (349,4)	A (424, 3)
I (338,3)	L (319,3)	* V (350,6)	G (425, 4)
I (339,1)	M (320,1)	* L (351,1)	T (426, 1)
		M (353,1)	
V (352,2)	V (336,4)		V (440, 2)
		C (369,1)	
S (413,1)	T (391,4)		
D (415,4)	D (393,5)		D (501, 5)
I (427, 4)	L (405,5)		I (513, 5)
I (428, 1)	V (406,1)		
D (429,2)	G (407,1)		D (515, 1)
R (430,2)	R (408,6)		R (516, 2)

(a,b), a, the site of putative binding-site residues; b, numbers in the 10 models; * Related with 4-coumaric acid; ** Related with ferulic acid.

Figure S1. Chemical structures of the six naturally occurring 4CL substrates, sinapic acid, 5-hydroxyferulate, ferulic acid, caffeic acid, 4-coumarate and trans-cinnamic acid. Acetate and propanoate was toward to acetyl-CoA and propanoyl-CoA by ACS. Succinylbenzoate was toward to Succinylbenzoyl-CoA by AAE.

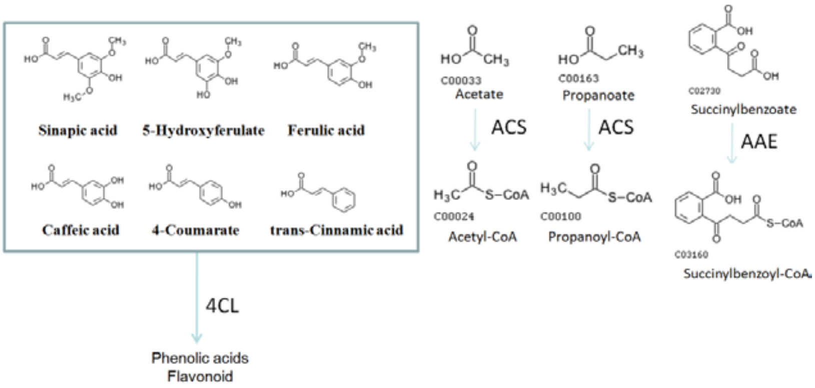

Figure S2. Phylogeny tree of AMP-binding enzyme (PF00501). A neighbor-joining tree containing 1207 sequences was generated based on the AMP-dependent synthetase/ligase domain sequences by Mega 5.02. A bootstrap value of 1000 replications was applied and all of sequences were classified into three clusters and 4 subgroups in the first cluster. Gene function of Arabidopsis thaliana in three clusters follow as: **1-1**, long chain acyl-CoA synthase; **1-2**, Acyl-acting anzyme/o-succinylbenzoate-CoA ligase/benzoate-CoA ligase; **1-3**, 4-coumarate:CoA ligase; **1-4**, acyl-CoA synthase/malony-CoA synthase; **2**, Acyl-acting anzyme/acetate-CoA ligase/AA-adenyl-dom(amino acid adenylation domain) protein; **3**, unknown.

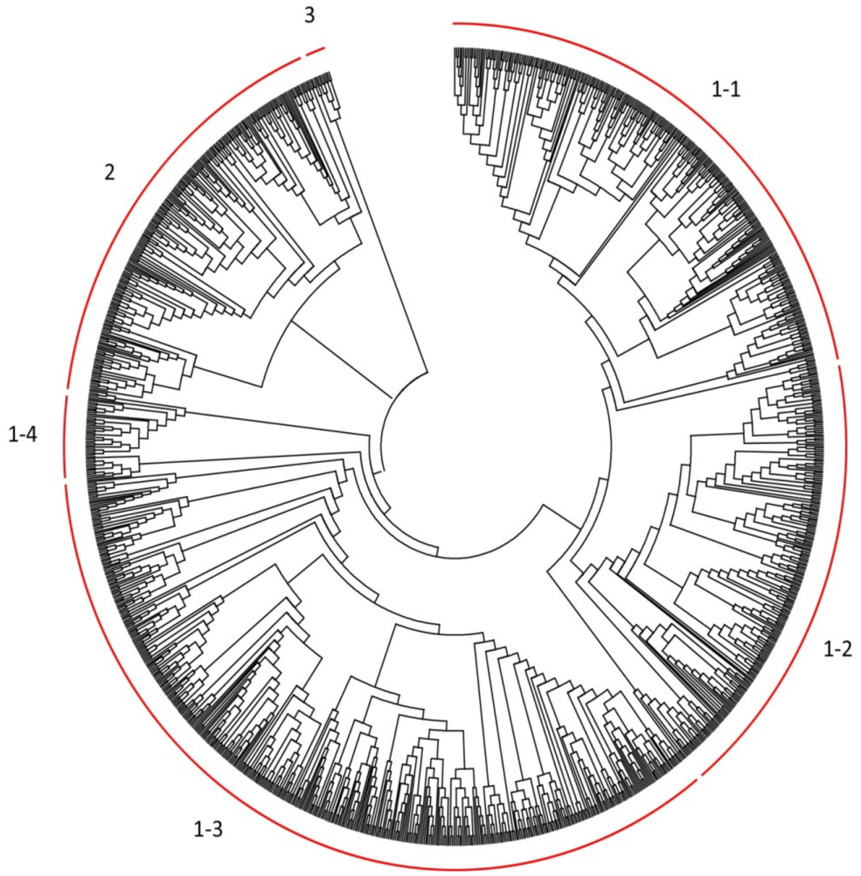

Figure S3. HPLC-MS analysis in bud of *Lonicera japonica* Thunb. (**A**,**B**) Chlorogenic acid(Retention time (RT) 13.20 min); (**C**) ferulic acid (RT 24.24 min); (**D**) Rutin(RT 24.24 min); (**E**) Hyperoside (RT 25.53 min), Isoquercitrin (RT 25.81 min); (**F**) Luteoloside (RT 25.39 min), quercitrin (RT 28.78 min); (**G**) luteolin (RT 37.78 min); (**H**) Quercetin (RT 38.19 min); (**I**) Apigenin(RT 43.19 min).

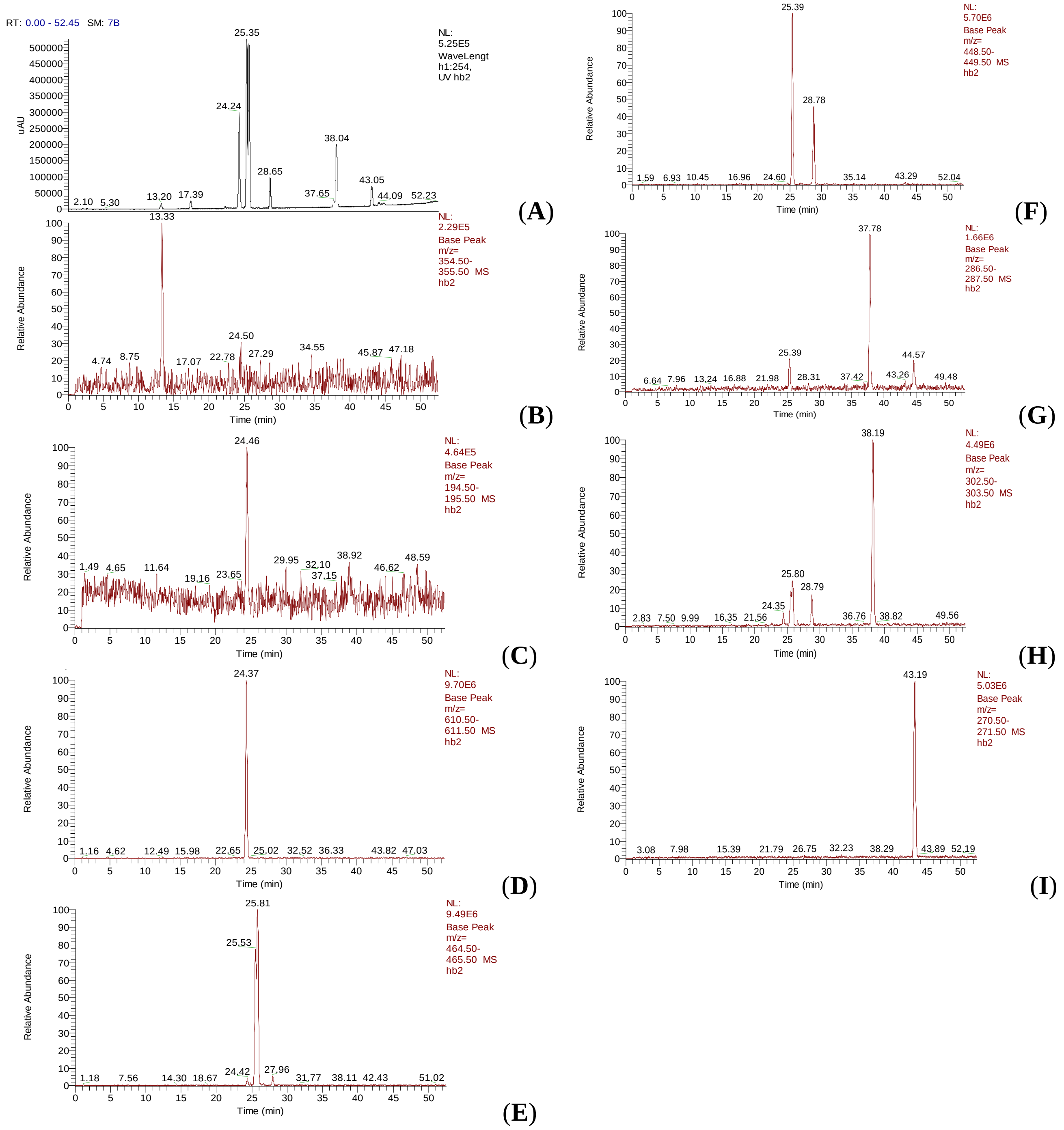

Figure S4. Phylogeny and expression of unknown protein sequences in Cluster2. A neighbor-joining tree containing 20 sequences was generated based on the AMP-dependent synthetase/ligase domain sequences. A bootstrap value of 1000 replications was applied. The rpkm value of sequences in flowers of Lonicera japonica Thunb is shown.

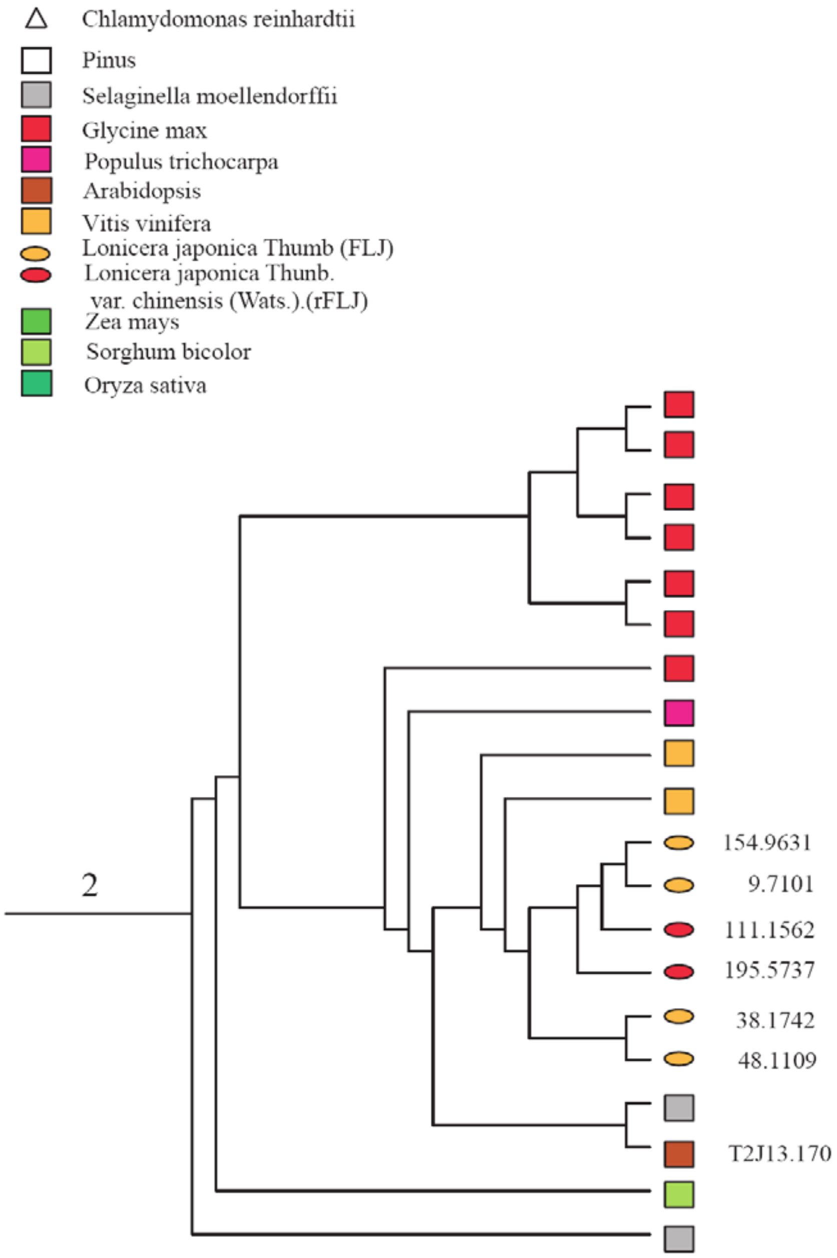

Figure S5. Phylogeny and expression of ACS sequences in Cluster1. A neighbor-joining tree containing 37 sequences was generated based on the AMP-dependent synthetase/ligase domain sequences. A bootstrap value of 1000 replications was applied. The rpkm value of sequences in flowers of Lonicera japonica Thunb is shown.

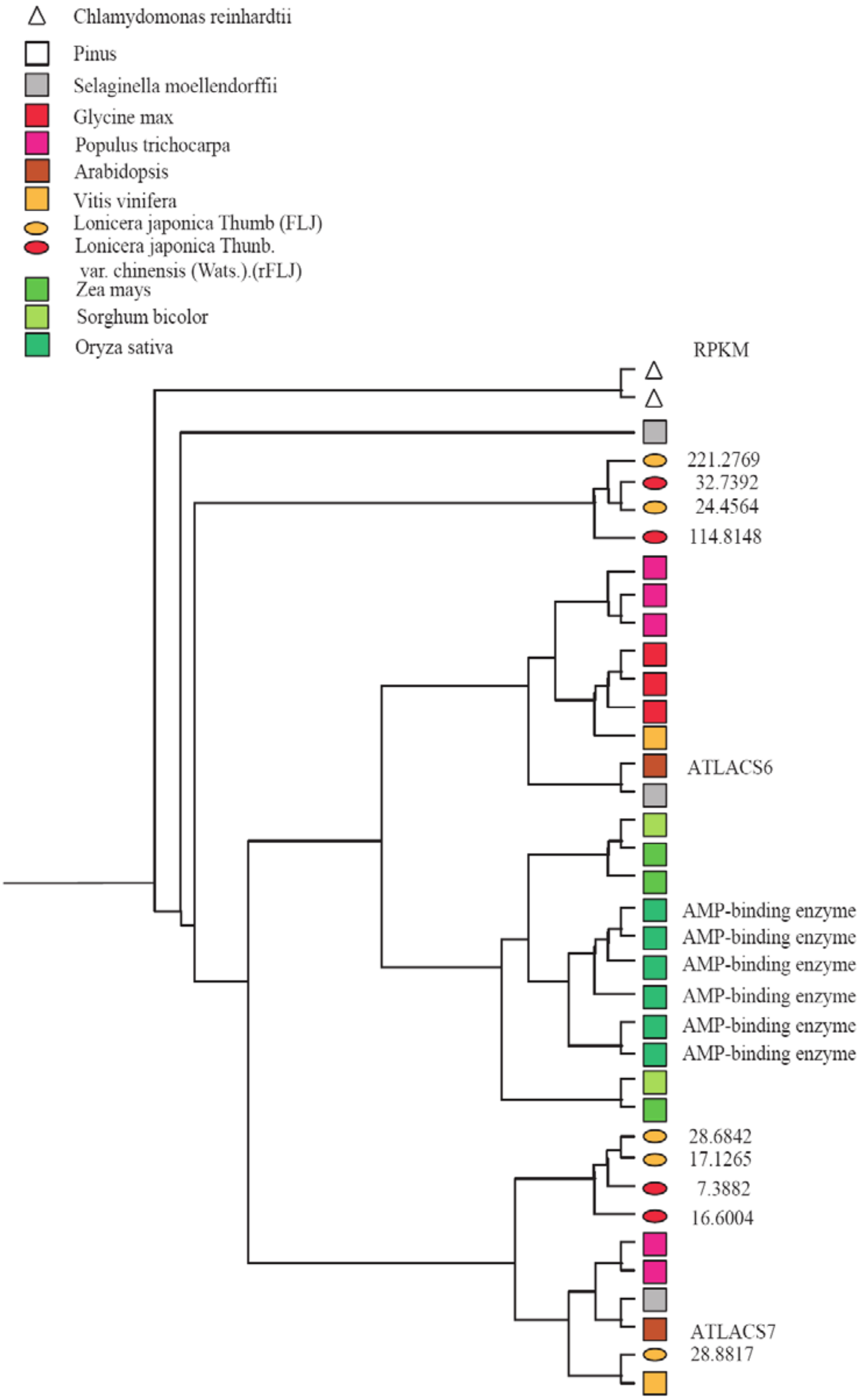

Figure S6. Phylogeny and expression of ACS sequences in Cluster4. A neighbor-joining tree containing 50 sequences was generated based on the AMP-dependent synthetase/ligase domain sequences. A bootstrap value of 1000 replications was applied. The rpkm value of sequences in flowers of Lonicera japonica Thunb is shown.

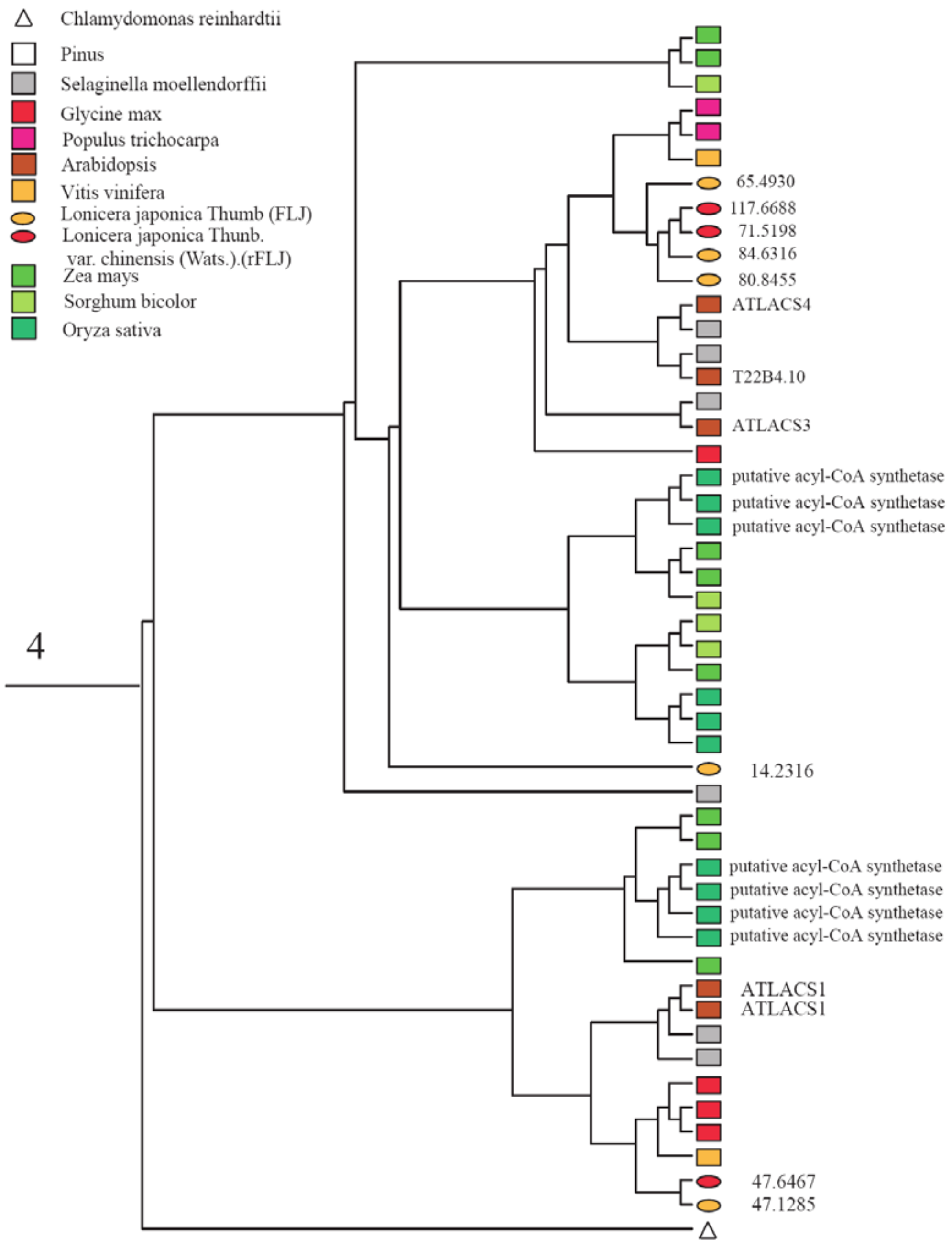

Figure S7. Protein structure of LJACS1, LJ4CL, LJAAE and LJACS2.

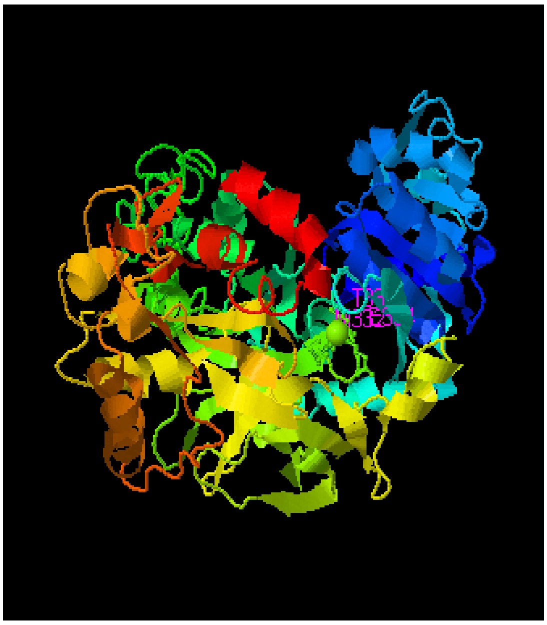

LJACS1 (Cluster1)

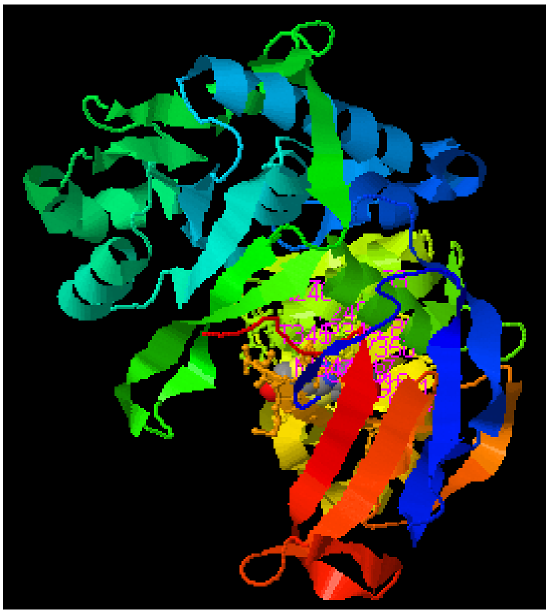

LJ4CL (Cluster3)

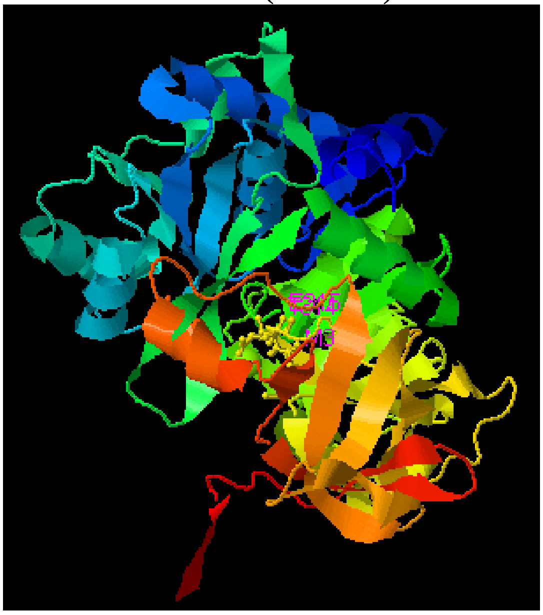

LJAAE (Cluster2)

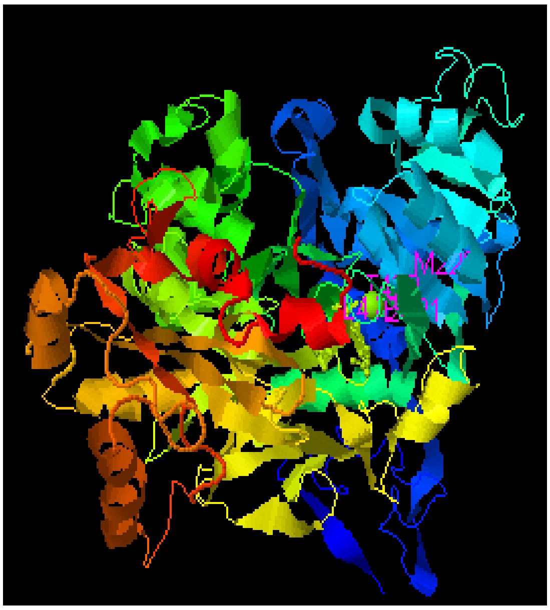

LJACS2 (Cluster4)

© 2014 by the authors; licensee MDPI, Basel, Switzerland. This article is an open access article distributed under the terms and conditions of the Creative Commons Attribution license (http://creativecommons.org/licenses/by/3.0/).
